# Feasibility Study of a Novel Transcatheter Tricuspid Annuloplasty System in a Porcine Model

**DOI:** 10.1016/j.jacbts.2022.02.022

**Published:** 2022-06-15

**Authors:** Wenzhi Pan, Yuliang Long, Xiaochun Zhang, Shasha Chen, Wei Li, Cuizhen Pan, Yingqiang Guo, Daxin Zhou, Junbo Ge

**Affiliations:** a Department of Cardiology, Zhongshan Hospital, Fudan University, Research Unit of Cardiovascular Techniques and Devices, Chinese Academy of Medical Sciences, Shanghai, China; b National Clinical Research Center for Interventional Medicine, Shanghai, China; c Department of Echocardiography, Fudan University, Zhongshan Hospital, Shanghai, China; d Department of Cardiovascular Surgery, Sichuan University, West China Hospital, Chengdu, China

**Keywords:** annuloplasty, transcatheter, tricuspid regurgitation, tricuspid valve, LVEF, left ventricular ejective fraction, RVEDD, right ventricular enddiastolic diameter, RVESD, right ventricular end systolic diameter, TR, tricuspid regurgitation

## Abstract

Gradually, more attention has been paid to tricuspid regurgitation. Formerly the “forgotten valvular heart disease,” it has been recognized as having high incidence and adverse prognosis if untreated. However, isolated tricuspid valve surgery carries substantial risk and is thus not recommended. The K-Clip transcatheter tricuspid annuloplasty system has recently emerged as a minimally invasive therapeutic option that may help address these unmet clinical needs. The K-Clip technology simulates the Kay’s procedure, which reduces the dimensions of the tricuspid annulus by folding and clamping the tricuspid annulus issue with a rigid clamping device. The feasibility of the K-Clip technology was investigated in this study, and annulus area reduction and declining tricuspid regurgitation grades were noted following the procedure. The findings of this study suggest that the K-Clip system may potentially be applicable as a novel transcatheter tricuspid annuloplasty device for the treatment of severe tricuspid in humans.

## Highlights

• The K-Clip transcatheter tricuspid annuloplasty system simulates the Kay’s procedure: shortening the distance between anterior leaflet and posterior-septal leaflets and resulting in a functionally bicuspid valve.
• The K-Clip system anchors on the annulus tissue using a corkscrew, with no need to cross the valve, and it is easy to adjust the corkscrew position by adjusting the depth and direction of 2 matched deflectable sheaths in the right atrium.
• Results from this study indicate that the K-Clip system may be potentially applicable in treatment of severe tricuspid regurgitation in humans.

## Visual Abstract

**Figure:**
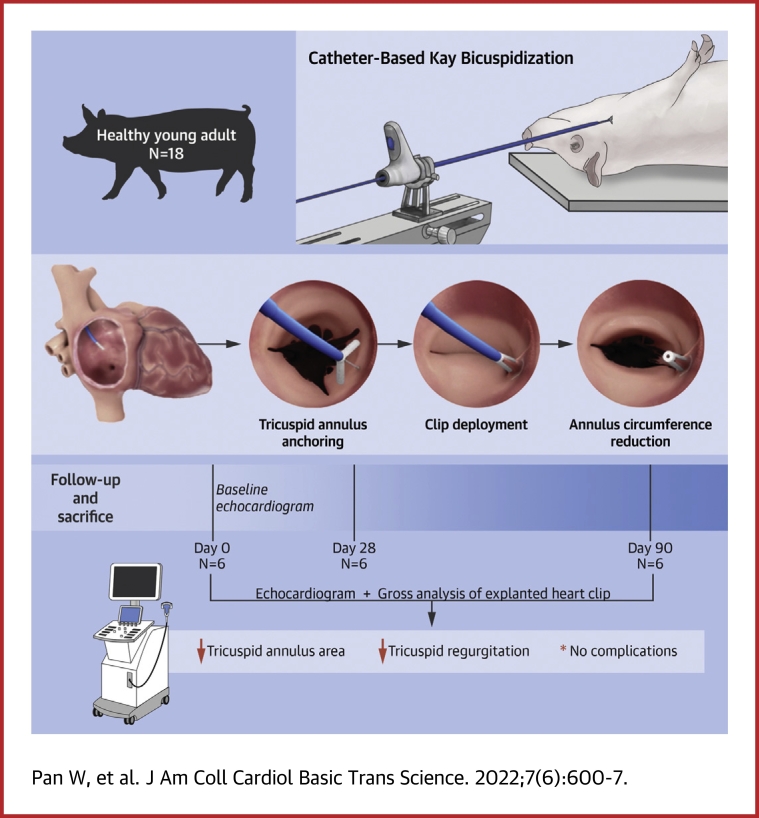

Gradually more attention has been paid to tricuspid regurgitation. Formerly the “forgotten valvular heart disease,” it has been recognized as having high incidence and adverse prognosis if untreated.^1^^,^^2^ Moreover, the majority of all tricuspid regurgitations are secondary or functional, which are mainly related to significant tricuspid annular dilatation.^1^ Surgical ring annuloplasty is the preferred treatment of secondary tricuspid regurgitation,^3^ and the Kay bicuspidization procedure, as one of classical surgical methods, has been evaluated as a safe and effective method with good mid and long-term results.^4^^,^^5^ However, isolated tricuspid valve surgery carries substantial risk and is thus not recommended, especially in those patients with earlier surgical history of left-sided valves.^6^^,^^7^ As an alternative to open-heart surgery, transcatheter tricuspid annuloplasty has emerged as a minimally invasive therapeutic option that may help address unmet clinical needs for patients with significant tricuspid annular dilatation but without indications of open-heart surgery.^3^ Mitralign (Mitralign Inc) is one of the few techniques based on the surgical mechanism that aims to shorten the dimensions of the tricuspid annulus,^8^ but until now, few data on its safety and feasibility have been available.

A novel transcatheter tricuspid annuloplasty system (K-Clip) simulating the Kay’s procedure was recently developed. The feasibility of the device has been tested in a porcine model. We first report the results of the feasibility study of this system.

## Methods

### Device description

**Figure 1:**
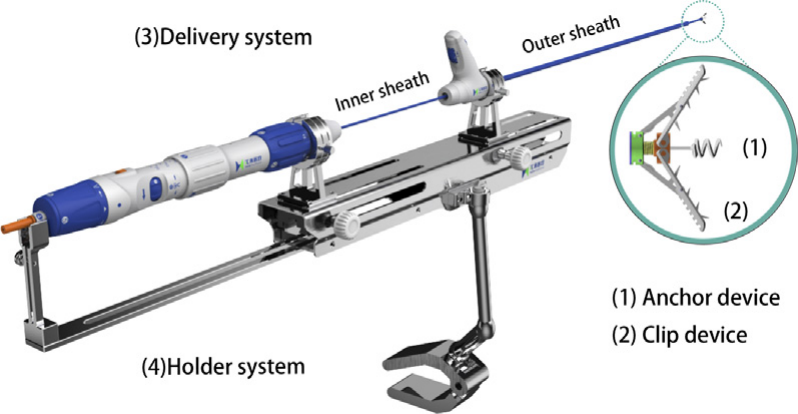
K-Clip System Illustration The K-Clip system consists of 4 major components: an anchor device, a clip device, a delivery system and a holder system.

The K-Clip tricuspid annuloplasty system (Huihe Medical Technology) consists of 4 devices (Video 1): 1) an anchor device; 2) a clip device; 3) a delivery system; and 4) a holder system (Figure 1). The anchor is composed of the corkscrew in the arm of the clip and the control handle inside of the delivery system. The length and the diameter of the corkscrew are 4.2 and 2.8 mm, respectively. The corkscrew can be screwed into the tricuspid annulus by thrusting and rotating the handle, and the maximum screwing depth is limited (≤4 mm) to avoid cardiac perforation. The clip includes 2 arms, and the tissue-facing surface of the 2 arms have teeth, preventing the tricuspid annulus tissue from slipping out. The maximum opening angle between 2 clip arms is 120°. A total of 4 kinds of clip sizes are available, with an arm length of 12, 14, 16, and 18 mm. The movement of the clamping arms, opening, closing, as well as detaching, is promoted by mechanical transmission, and the control system is loaded on the delivery system. The delivery system is composed of 2 matched deflectable sheaths: the outer sheath (18-F), with the bending angles in the range of 0-45°; and the inner sheath (15-F), with the bending angles in the range of −90° to 90°. After the inner sheath passes through the center hole of the outer sheath, the delivery system can achieve greater dynamic bending range. The anchor and the clip are deployed on the distal end of the inner sheath, with their control systems on the handle bar on the proximal end of the inner sheath. The whole delivery system is fastened to the adapters of the holder system during the procedure. The 2 sheaths’ forward and backward movements can be controlled separately and precisely through the 2 rotations of the holder system, so that the 2 sheaths’ rotatable or fixed situation can be switched using the knob of the adapters.

### Animal model

Between September 2020 and October 2020, 18 young adult swine (Yorkshire pigs) weighing 80 ± 5 kg were enrolled in the study. All animals were studied as an acute model (6 pigs, sacrificed immediately after the procedure), subacute model (6 pigs, sacrificed at the 28th day after the procedure), and chronic model (6 pigs, sacrificed at the 90th day after the procedure). Warfarin (0.75 mg) was given orally after the procedure until the endpoint. Low-molecular-weight heparin sodium (Fraxiparine 4,100 international units twice per day subcutaneously) was injected for 3 days after the procedure. Morphine hydrochloride injection (10 mg) was intramuscularly injected per day for the first 3 days after the operation, and after that ibuprofen (0.2 g twice per day) was given orally until 1 week after the operation. All animals received humane care in compliance with the 2011 “Guide for the Care and Use of Laboratory Animals.”

### Procedure

**Figure 2:**
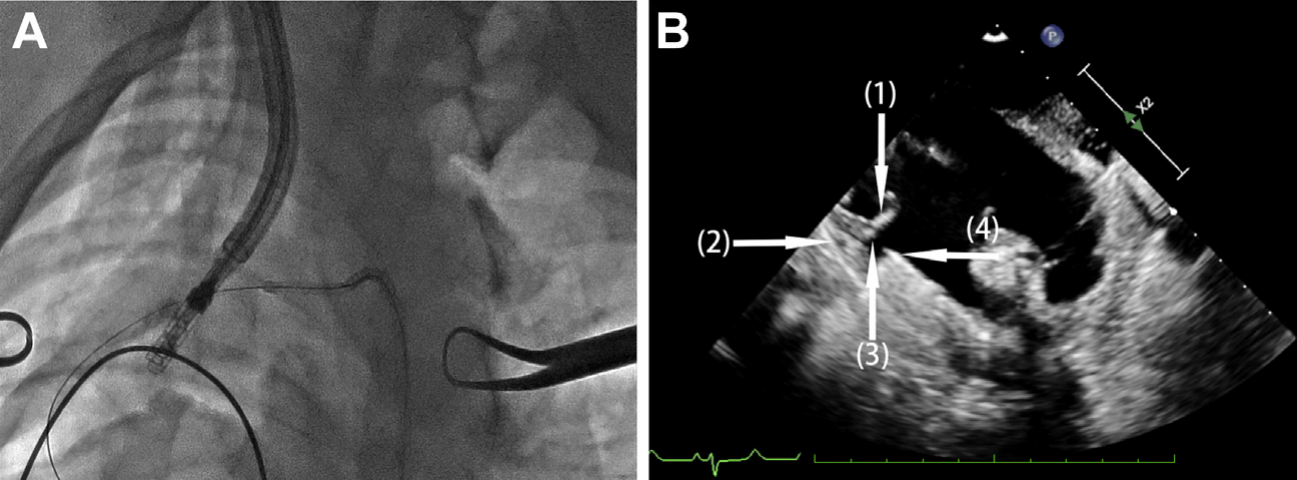
Fluoroscopy and Direct Epicardial Echocardiogram of Annulus Anchoring Procedure **(A)** The guidewire placed in the right coronary artery in help with identifying the location of the tricuspid annulus on fluoroscopy. **(B)** In the apical 2-chamber view of echocardiogram, **white arrows** indicate **(1)** leaflet issue; **(2)** right coronary artery; **(3)** tricuspid annulus; and **(4)** the distal end of the inner sheath.

**Figure 3:**
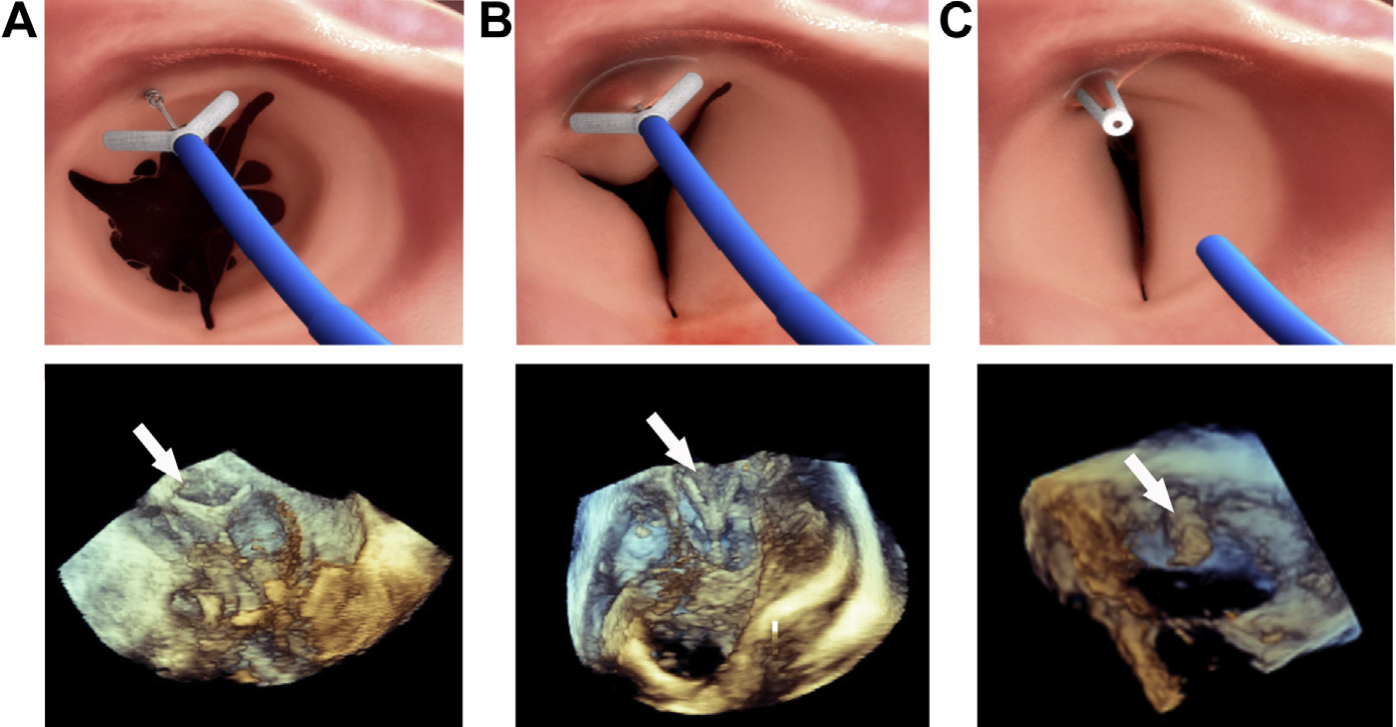
Simulation and Echocardiographic Images Illustrating the K-Clip Procedure **(A)** The device is anchored on the annulus using a corkscrew. **(B)** The clip shortens the circumference of the annulus resulting in a functionally bicuspid valve. **(C)** The clip is detached from the delivery system. The **arrows** are pointing to the clip.

Heparin was administered before the procedure to maintain an activated clotting time of 300-600 seconds. The procedure was performed under general anesthesia with fluoroscopic and direct epicardial echocardiographic guidance. The echo probe was directly placed on the epicardium to reproduce 2- and 3-dimensional images in the standard views, including the X-plane of tricuspid annulus, the short- and long-axis views, and apical 2- and 4-chamber views. Before the clip deployment, a guidewire was placed in the right coronary artery to help with identifying its location relative to the tricuspid annulus on fluoroscopy. The outer sheath was introduced into the right atrium through the right jugular vein; afterward, the inner sheath was loaded with the anchor, and the clip was inserted into the outer sheath. The position of the distal end of the delivery sheath was adjusted so that the corkscrew was parallel to the annulus and pointing to the anterior and posterior tricuspid leaflet commissures. Under the guidance of direct epicardial echocardiographic imaging and fluoroscopy, the corkscrew was drilled into the annulus between the leaflet and right coronary artery in the apical 2- chamber view (Figure 2). Then, 2 clip arms were unfolded slowly and moved toward the annulus (Figure 3A). The anchor was withdrawn to keep the annulus issue in contact with the clip arms. Afterward, the clip arms were closed, and then the circumference of the annulus was reduced (Figure 3B). The stability of the clip, the reduction of tricuspid annular area, and the transvalvular gradient were evaluated, and a repeat right coronary arterial angiogram was obtained to exclude obvious coronary stenosis. If the clamping was satisfactory, the clip was detached from the delivery sheath (Figure 3C). The delivery system (outer and inner sheath), the coronary guide wire, and angiography catheter were pulled out.

### Evaluation protocol

During the procedure, the rates of acute procedure success and complications were analyzed. Acute procedural success was defined as device clamping of the tricuspid annulus and successful withdrawal of the delivery system, while not causing tricuspid valve dysfunction and coronary stenosis. A detailed 2-dimensional and Doppler echocardiographic examination was performed at baseline and immediately after, on the 28th day, and on the 90th day after the procedure.

The animals were sacrificed according to the protocol. Postmortem, the right atrium and right ventricle were opened and the tricuspid annulus was inspected to evaluate the clip placement, tissue damage, device surface endothelialization, and thrombosis. In addition, the heart, liver, spleen, lung, kidney, and brain of the animal were removed for gross observation, and pathological sections were used to observe thromboembolism and tissue change.

### Statistical analysis

Continuous variables are expressed as mean ± SD. Categorical variables are reported as frequencies and percentages. Normal distribution was assessed by the Kolmogorov-Smirnov test. Comparisons of continuous variables between baseline and postprocedure were compared with the paired Student's *t*-test. Comparisons of tricuspid regurgitation grades among different time points were performed using nonparametric Friedman's test. A 2-sided *P* value <0.05 was considered statistically significant. Statistical analyses were performed using SPSS version 19 (IBM Corp).

## Results

### The acute results

**Figure 4:**
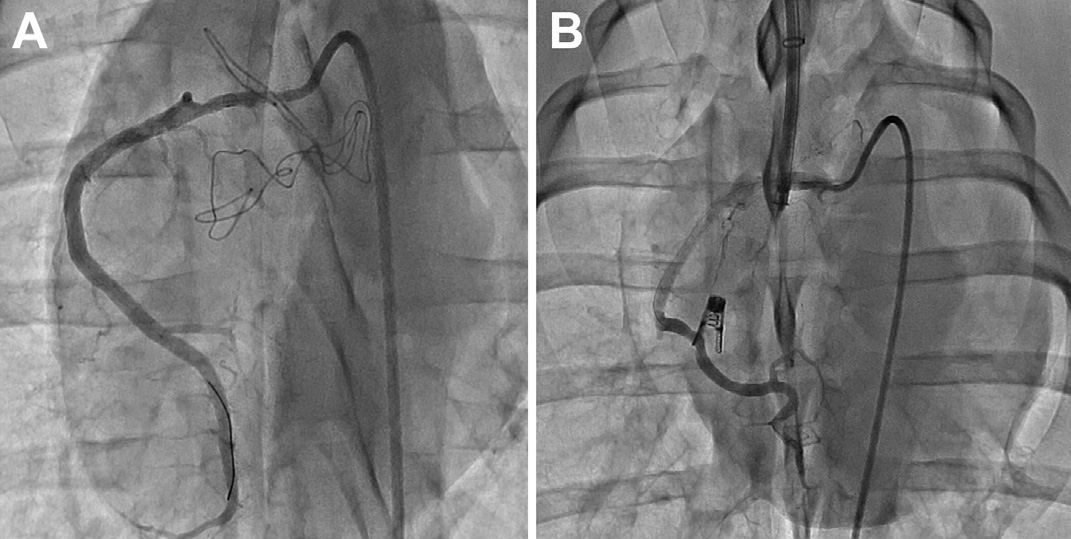
Right Coronary Angiogram Angiogram before **(A)** and after **(B)** the K-Clip procedure showing good opacification of the coronary arteries without obvious coronary stenosis.

A total of 18 pigs were included in the study. All of the pigs were male, and their mean weight was 81.29 ± 1.27 kg. All animals were implanted with the K-clip device (12 mm), and the acute procedure success rate was 100%. The total procedure time (skin to skin, ie, from skin puncturing to skin suturing) was 23.67 ± 4.21 min. Repeat coronary angiogram has excluded coronary stenosis after the clip implantation (Figure 4). There was no bleeding, cardiac perforation, pericardial effusion or any other procedure-related complication. All animals reached the corresponding end point, and no operation related severe complication was noted.

Of 13 pigs with baseline tricuspid regurgitation grade 1+/2+, 9 pigs (69.2%) experienced a decline of 1 grade, and 3 pigs (23.1%) experienced a decline of 2 grades. Immediate repeat echocardiogram also revealed a rapid significant reduction in annular area (from 10.16 ± 1.45 mm^2^ to 8.97 ± 1.15 mm^2^, a reduction of 11.7%; *P =* 0.010). The right cardiac dimensions were also on the decline, but without significant difference: right atrium anteroposterior diameter (from 29.72 ± 4.18 mm to 28.89 ± 3.88 mm; *P =* 0.540), right ventricular end-diastolic diameter (from 27.94 ± 3.62 mm to 26.67 ± 3.33 mm; *P =* 0.256), and right ventricular end-systolic diameter (from 18.33 ± 3.24 mm to 17.56 ± 3.29 mm; *P =* 0.500). Also, no statistical difference has been measured in both maximum and mean transvalvular gradient and left ventricular ejection fraction before and after the procedure (Table 1).

**Table 1:** Cardiac-Size, Hemodynamic, and Functional Changes After the K-Clip Procedure (N = 18)

	Baseline	After Procedure	*P* Valve
Tricuspid annular area, mm^2^	10.16 ± 1.45	8.97 ± 1.15	0.010
Right atrium anteroposterior diameter, mm	29.72 ± 4.18	28.89 ± 3.88	0.540
Right ventricular end diastolic diameter, mm	27.94 ± 3.62	26.67 ± 3.33	0.256
Right ventricular end systolic diameter, mm	18.33 ± 3.24	17.56 ± 3.29	0.500
Maximum transvalvular gradient, mm Hg	1.0 ± 0.0	1.0 ± 0.0	—
Mean transvalvular gradient, mm Hg	0.67 ± 0.49	0.94 ± 0.24	0.063
Left ventricular ejection fraction, %	60.89 ± 2.72	60.44 ± 2.91	0.639

### Follow-up results

The results of the echocardiographic evaluations of tricuspid valve function immediately after implantation on the 28th day and at the time of explantation are given in Table 2. No pig had a mean transvalvular gradient >1 mm Hg. One pig developed minor regurgitation at the time of explantation. There was significant difference of tricuspid grades *(P =* 0.020) among different time points, which indicated the feasibility of the device to treat tricuspid regurgitation. Both maximum and mean transvalvular gradient were no more than 1 mm Hg.

**Table 2:** Echocardiographic Evaluations of Tricuspid Valve Function Immediately After Implantation, in the Middle Term, and at the Time of Explantation

	Baseline	Immediately After	28 days	90 days
Pig 1	0/1	0/1	0/1	0/0
Pig 2	2+/1	2+/1	1+/0	1+/0
Pig 3	1+/0	0/1	0/1	0/1
Pig 4	1+/1	1+/1	1+/0	1+/1
Pig 5	2+/1	1+/1	1+/1	1+/1
Pig 6	2+/0	0/1	0/1	1+/1

Values are MR grades/mean transvalvular gradient (mm Hg).

### Explantation results

In total, 6 pigs were explanted immediately after the procedure, 6 pigs were explanted at the 28th day after the procedure, and the other 6 pigs lived to the endpoint and were then sacrificed.

**Figure 5:**
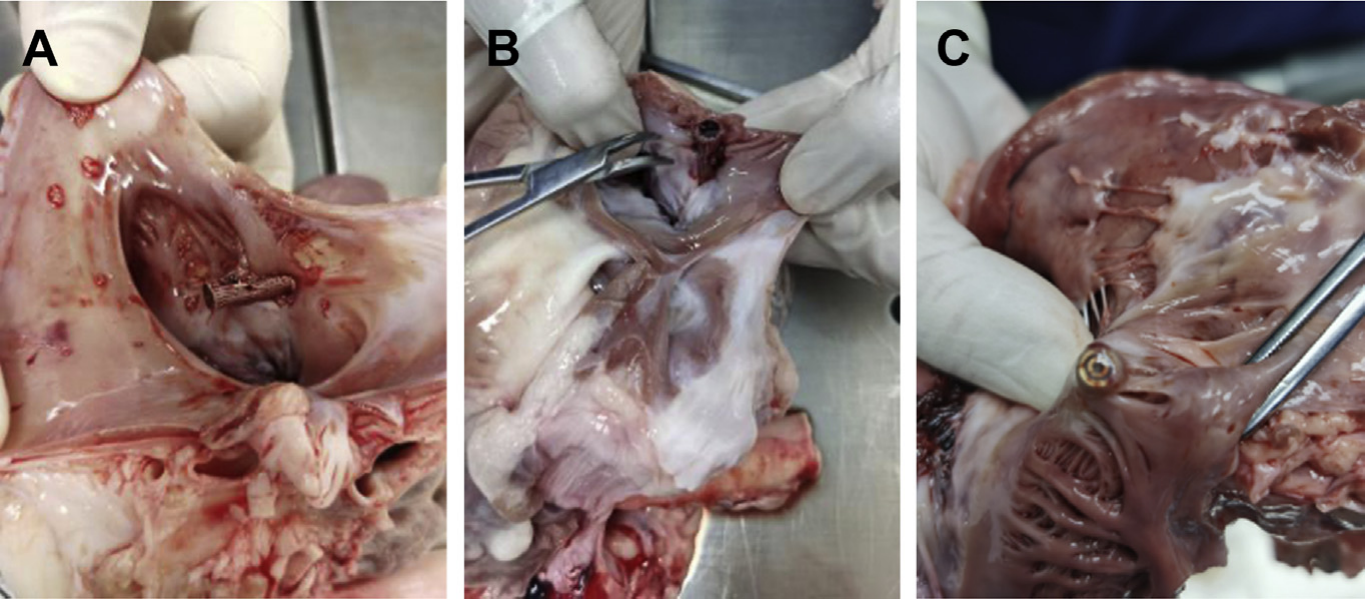
The Gross Observation of the Heart Postmortem Shown are animals explanted **(A)** immediately after the procedure and **(B)** 28 days after the procedure, and the clip clamped the tricuspid annulus issue. **(C)** Shown is an animal explanted at the 90th day after the procedure. The clip device has been completely endothelialized.

In the acute test, the gross observation showed that the clip device was securely attached to the tricuspid annulus tissue with annulus tissue in between the clip arms (Figure 5A). In the subacute test and endpoint explantation, gross observation showed that the clip device had been completely endothelialized (Figures 5B and 5C). There was no cardiac perforation and no impairment of the tricuspid valve complex. None of the 12 pigs developed infective endocarditis. The heart, liver, spleen, kidneys, and brain were unremarkable in terms of thrombosis, thromboembolism, and infarcts in all animals.

## Discussion

The K-Clip system is a novel transcatheter tricuspid annuloplasty system, and this short-term follow-up animal study demonstrated that it was feasible and safe to use this system to perform a catheter-mediated tricuspid valve annuloplasty in a porcine model. Follow-up echocardiograms showed no obvious tricuspid valve dysfunction. The explantation results showed no device detachment, no cardiac perforation, and no impairment of the tricuspid valve complex. Full device endothelialization was observed on the 90th day after the procedure. The feasibility of the device to treat tricuspid regurgitation was confirmed by a remarkable decline of tricuspid regurgitation grades after the procedure.

Because of the high risk of isolated tricuspid surgical repair, especially in those patients with a history of left-side valve surgery,^6^^,^^7^ there is an urgent need of less invasive techniques in treatment of severe tricuspid regurgitation. Recently, several transcatheter therapies have emerged, but until now, none of them has been approved to be used commercially.^8^, ^9^, ^10^

The principle of the K-Clip system was based on the Kay’s annuloplasty: reducing the size of annulus by a figure-eight suture plication of the posterior leaflet, shortening the distance between anterior leaflet and posterior-septal leaflets, and resulting in a functionally bicuspid valve.^11^ The Trialign system (Mitralign, Inc) is another transcatheter annuloplasty system that mimics the Kay surgical procedure, which has finished a small amount of clinical application.^8^^,^^12^ According to the early feasibility study of the Trialign system, a 6.5% reduction of tricuspid annulus area has proven to be effective in tricuspid regurgitation correction.^12^ An 11.7% reduction of tricuspid annulus area has been evaluated in this study; therefore, the principle of the K-Clip may potentially be applicable in the treatment of severe tricuspid regurgitation in humans. Both systems are delivered via percutaneous jugular vein access, and the route is short and straight for the convenience of manipulation. However, compared with Trialign, the K-Clip has its theoretical advantages.

First, the K-clip procedure may be technically easier than that of Trialign. The Trialign system needs to fasten 2 pledgets on the annulus, which need to manipulate the delivery catheter advance across the tricuspid valve, and penetrates the leaflet tissue using a wire from right ventricle to right atrium; afterwards, the wire needs to be snared from the jugular vein using the second sheath and externalized. Catheter navigation in the enlarged right heart chambers can be challenging, and this process needs to be repeated at least twice.^12^, ^13^, ^14^ The K-Clip system, however, is anchored directly on the annulus tissue with the corkscrew, with no need to cross the valve, and it is easy to adjust the corkscrew position by adjusting the depth and direction of 2 matched deflectable sheaths in the right atrium under the guidance of echocardiogram and fluoroscopy. Because the K-Clip system is easily manipulated, the catheter manipulation time of K-Clip procedure in this study was just 23.67 ± 4.21 minutes, which was much less than that previously reported with Trialign (total fluoroscopy time: 96.2 ± 30.8 minutes).^12^

Second, the K-clip procedure may have a higher safety coefficient than that of Trialign. In the K-clip procedure, anchoring on the annulus using the corkscrew rather than puncturing the leaflet tissue can effectively avoid procedure-related risks, such as pledget detachment, leaflet impairment, coronary artery rupture, cardiac perforation, and the like.^12^^,^^14^ Also, anchoring with the corkscrew is very reliable, and no device detachment has been noted in this study. Moreover, the depth of the corkscrew is limited to 4 mm to reduce the risk of cardiac perforation.

Finally, the K-clip procedure may have higher flexibility than that of Trialign. In the K-clip procedure, if the site of the anchor on the annulus is not satisfactory, the corkscrew can be withdrawn easily; afterward, the position of the corkscrew can be readjusted. However, in the Trialign procedure, after the wire passes through the leaflet tissue, changing the position of the pledget cannot be that easy.

The first-in-man studies of K-Clip system are now underway in China.

### Study limitations

First, the study follow-up was only 90 days, and no long-term follow-up results were given. Second, this was an animal study that was performed in healthy animals. Although annulus area reduction and declining tricuspid regurgitation grades after the procedure have been noted, the clinical efficacy of the procedure in treatment of severe tricuspid regurgitation could not be tested reliably in this model, and clinical trials will be necessary to fully evaluate this device. Finally, this study only tested the feasibility of 1 clip implantation; in patients with remarkable dilated annulus, more than 1 clip can be implanted to reinforce effects of annulus reduction.

## Conclusions

K-Clip is an easy-to-operate transcatheter annuloplasty system, which has been proved feasible and safe in catheter-mediated tricuspid valve repair in a porcine model. The K-Clip system may potentially be applicable as a novel transcatheter tricuspid annuloplasty device to assist patients with severe tricuspid regurgitation who are at high risk of undergoing surgery.

Perspectives **COMPETENCY IN MEDICAL KNOWLEDGE:** Tricuspid annuloplasty is the cornerstone of surgical treatment for functional tricuspid regurgitation, with the goal of improving leaflet coaptation by correcting annular dilation and restoring annular geometry. Suture and ring annuloplasty procedures are the 2 main surgical methods utilized to accomplish tricuspid annuloplasty. Suture annuloplasty reduces the size of the tricuspid annulus by cinching it with a continuous suture. The majority of suture annuloplasty procedures are modified variants of the Kay bicuspidization and involve plication of both the posterior and anterior annuli. Suture annuloplasty procedures have the benefit of being technically simple and quick to perform. Furthermore, unlike ring annuloplasty, suture annuloplasty does not require a prosthetic implant, and the risk of postoperative conduction problems is minimal. K-clip is different from the above 2 technologies. When compared with existing suture annuloplasty technologies, the K-Clip technique is more convenient to employ because it does not require the device to pass through the tricuspid valve complex to be fastened, and it preserves the advantage of suture annuloplasty technology's fewer implants. In addition, the K-Clip system uses a clip rather than a suture to fold the tricuspid annulus. Because the clip arm has a significantly larger surface area than the suture, it can relieve pressure on the myocardium's surface and prevent suture damage.

**TRANSLATIONAL OUTLOOK:** Future clinical studies should explore the feasibility, safety, as well as short- and long-term efficacy of the K-Clip technology in the treatment of severe tricuspid regurgitation.

## Funding Support and Author Disclosures

This study is supported by National Key R&D Program of China (2020YFC2008100) and Shanghai Engineering Research Center of Interventional Medicine (19DZ2250300). The authors have reported that they have no relationships relevant to the contents of this paper to disclose.

## Appendix

Supplemental Video 1 The illustration of the K-clip technology. The K-Clip technology simulates the Kay’s procedure, which reduces the dimensions of the tricuspid annulus by folding and clamping the tricuspid annulus issue with a rigid clamping device.
